# A theory-based process evaluation alongside a randomised controlled trial of printed educational messages to increase primary care physicians’ prescription of thiazide diuretics for hypertension [ISRCTN72772651]

**DOI:** 10.1186/s13012-016-0485-4

**Published:** 2016-09-13

**Authors:** Justin Presseau, Jeremy M. Grimshaw, Jacqueline M. Tetroe, Martin P. Eccles, Jill J. Francis, Gaston Godin, Ian D. Graham, Janet E. Hux, Marie Johnston, France Légaré, Louise Lemyre, Nicole Robinson, Merrick Zwarenstein

**Affiliations:** 1 Ottawa Hospital Research Institute, The Ottawa Hospital, General Campus, 501 Smyth Road, Box 201B, Ottawa, Ontario K1H 8L6 Canada; 2 School of Epidemiology, Public Health and Preventive Medicine, 451 Smyth Road, Ottawa, Ontario K1H 8M5 Canada; 3 Department of Medicine, University of Ottawa, 451 Smyth Rd, Ottawa, Ontario K1H 8M5 Canada; 4 Retired, Ottawa, Canada; 5 Institute of Health and Society, Newcastle University, Baddiley-Clark Building, Richardson Road, Newcastle Upon Tyne, NE2 4AX England; 6 School of Health Sciences, City University London, Northampton Square, London, EC1V 0HB UK; 7 Faculty of Nursing, Laval University, Pavillon Ferdinand-Vandry, 1050 Avenue de la Medicine, Room 1445, Quebec City, Quebec G1V 0A6 Canada; 8 School of Nursing, Faculty of Health Sciences, University of Ottawa, 451 Smyth Rd, Ottawa, Ontario K1H 8M5 Canada; 9 Canadian Diabetes Association, 522 University Ave, Toronto, ON M5G 2A2 Canada; 10 Institute of Applied Health Sciences, College of Life Sciences and Medicine, 2nd floor, Health Sciences Building, Foresterhill, Aberdeen, AB25 2ZD UK; 11 Department of Family Medicine and Emergency Medicine, Université Laval, Québec City, Québec G1K 7P4 Canada; 12 School of Psychology, University of Ottawa, 120 University, Social Sciences Building FSS-5052, Ottawa, Ontario K1N 6N5 Canada; 13 Centre for Studies in Family Medicine, Department of Family Medicine, Schulich School of Medicine and Dentistry, Western University, 1465 Richmond Street, London, Ontario N6A 3K7 Canada; 14 Institute for Clinical Evaluative Sciences, University of Toronto, 2075 Bayview Avenue, Toronto, Ontario M4N 3M5 Canada

## Abstract

**Background:**

Pragmatic trials of implementation interventions focus on evaluating whether an intervention changes professional behaviour under real-world conditions rather than investigating the mechanism through which change occurs. Theory-based process evaluations conducted alongside pragmatic randomised trials address this by assessing whether the intervention changes theoretical constructs proposed to mediate change. The Ontario Printed Educational Materials (PEM) cluster trial was designed to increase family physicians’ guideline-recommended prescription of thiazide diuretics. The trial found no intervention effect. Using the theory of planned behaviour (TPB), we hypothesised that changes in thiazide prescribing would be reflected in changes in intention, consistent with changes in attitude and subjective norm, with no change to their perceived behavioural control (PBC), and tested this alongside the RCT.

**Methods:**

We developed and sent TPB postal questionnaires to a random sub-sample of family physicians in each trial arm 2 months before and 6 months after dissemination of the PEMs. We used analysis of covariance to test for group differences using a 2 × 3 factorial design. We content-analysed an open-ended question about perceived barriers to thiazide prescription. Using control group data, we tested whether baseline measures of TPB constructs predicted self-reported thiazide prescribing at follow-up.

**Results:**

Four hundred twenty-six physicians completed pre- and post-intervention questionnaires. Baseline scores on measures of TPB constructs were high: intention mean = 5.9 out of 7 (SD = 1.4), attitude mean = 5.8 (SD = 1.1), subjective norm mean = 5.8 (SD = 1.1) and PBC mean = 6.2 (SD = 1.0). The arms did not significantly differ post-intervention on any of the theory-based constructs, suggesting a possible ceiling effect. Content analysis of perceived barriers suggested post-intentional barriers to prescribing thiazides most often focused on specific patient clinical characteristics and potential side effects. Baseline intention (*β* = 0.63, *p* < 0.01) but not PBC (*β* = 0.04, *p* = 0.78) predicted 42.6 % of the variance in self-reported behaviour at follow-up in the control group.

**Conclusions:**

Congruent with the Ontario Printed Educational Messages trial results and aligned with the TPB, we saw no impact of the intervention on any TPB constructs. The theoretical basis of this evaluation suggests possible explanations for the failure of the PEM intervention to change professional behaviour, which can directly inform the design and content of future theory-based PEM interventions to change professional behaviour.

**Trial registration:**

ISRCTN, Canada ISRCTN72772651

**Electronic supplementary material:**

The online version of this article (doi:10.1186/s13012-016-0485-4) contains supplementary material, which is available to authorized users.

## Background

Hypertension is widespread. In the province of Ontario in Canada, 21 % of the adult population has an elevated blood pressure, with prevalence rising as a function of age to 52 % in those aged 60–79 [1]. Rates in Ontario are largely consistent with prevalence rates across Canada as a whole [2]. While numerous hypertension medication options are available for managing hypertension, thiazide diuretics are among the most well tolerated [3], have cardiovascular protective effects [4] and have been consistently recommended as first-line agents in clinical practice guidelines for managing uncomplicated hypertension [5, 6]. They are also the least expensive and, if more widely used, would result in substantial annual savings if used over more expensive drug options [7]. In spite of this, thiazides are not prescribed as often as other antihypertensive drugs [8].

Reviews of disseminating printed educational materials (PEMs) suggest that they can be effective in promoting health professional behaviour change in some instances but not in all, and there is wide variation in effectiveness and methodological rigour between existing randomised controlled trials (RCTs) [9, 10]. The large factorial cluster randomised Ontario Printed Educational Messages (OPEM) trial (and associated TRY-ME sub-trial) for promoting thiazide diuretic prescription was designed to address the limitations of previous trials [11]. This trial tested the effectiveness of short and long educational messages (in this case, PEMs) for increasing thiazide prescription to elderly patients with uncomplicated hypertension, a recommended antihypertensive drug that is at least as effective as other classes of antihypertensive at reducing morbidity and mortality while being less expensive. The trial found *no evidence* that PEMs increased the number of patients receiving thiazide diuretics. While the size and rigour of the trial provide convincing evidence that the PEMs were not effective for changing this clinical behaviour, the trial was not designed to investigate the reason for this lack of effect. There is a need to better understand the possible mechanisms that mediate intervention effects in RCTs of implementation interventions to gain insight into how effective interventions change behaviour and why ineffective interventions do not. A challenge for implementation researchers is to develop methods for exploring these causal mechanisms alongside rigorous tests of implementation interventions.

There is increasing recognition of the value of process evaluations alongside trials of complex interventions such as professional behaviour change interventions [12–14]. Process evaluations complement outcome evaluation by investigating how an intervention may work; how it is delivered, the mechanisms through which effects may operate and its contextual moderators [12]. Process evaluations can offer robust explanations of why an intervention fails to improve health care (or even does harm) by assessing whether or not the intervention changes the proposed mediators of improved outcomes. Process evaluations often involve the ad hoc selection of context-specific indicators of process and use quantitative and/or qualitative methods to provide a detailed assessment of processes rooted in the context of the trial. Rather than ad hoc selection of process indicators, selecting indicators informed by theories of behaviour is an arguably superior approach to understand the determinants of the outcome. In turn, this could increase the ability to generalise findings to other clinical problems, professional groups and settings.

Behavioural science has systematically operationalized theories concerning determinants of behaviour and how they are associated with each other. This may be useful for understanding the mechanisms underlying implementation interventions designed to change clinicians’ behaviour [15]. Such theories employ standard definitions of constructs and measurement methods, which may be useful for exploring causal mechanisms of implementation interventions by testing whether intervention effects operate via hypothesised mediating pathways. Theory-based process evaluations can therefore contribute to the accumulation of a knowledge base of how implementation interventions might operate [16].

Using theory to explore mediating mechanisms of behaviour change interventions is commonplace in some fields [17, 18] and shows promise for greater use in exploring the mechanisms of action in implementation interventions where healthcare professional behaviour change is involved. For example, Ramsay and colleagues [19] conducted a post-intervention theory-based process evaluation of two interventions aiming to reduce inappropriate test-ordering evaluated within a randomised trial. The process evaluation focused upon investigating the causal mechanisms of the intervention for three of the targeted tests. They showed that behavioural intention partially mediated the intervention effect in two of the three tests assessed and suggested that the lack of mediation for the third test may have partly been an function of a ceiling effect on intention. Hrisos and colleagues conducted a theory-based process evaluation alongside an intervention designed to change physicians’ intentions and found that a persuasive communications intervention was mediated by theory-based constructs (attitude and subjective norm) [20]. These examples demonstrate the utility of drawing upon behavioural theory to hypothesise and test the mediating mechanisms of interventions for promoting health professional behaviour change.

When interventions are explicitly theory-based, the selection of a particular theory upon which to base the process evaluation is straightforward and can explicitly tie the intervention to potential mediating pathways [14, 21]. However, many implementation interventions are designed pragmatically without an explicit theoretical basis but likely involve an implicit model of how the intervention may change clinicians’ behaviour [15]. Such implicit models can be to some extent reverse-engineered by examining the description of the intervention content, which provides an indication of the factors the intervention designers assumed needed to change. Thus, with sufficient intervention description, implicit models can be mapped onto theoretical constructs that are likely to be changed [22, 23]. Linking these constructs back to a theoretical model that includes such constructs provides a basis for assessing mediating mechanisms through which the intervention effects can be hypothesised to operate regardless of whether the intervention itself is explicitly theory-based.

**Fig. 1:**
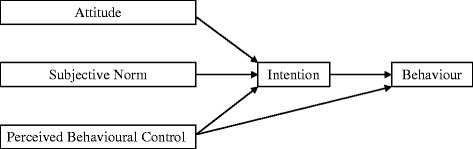
The theory of planned behaviour [24]

The theory of planned behaviour [24] (TPB) is a social cognition model of behaviour with well evidenced predictive utility across a number of populations and behaviours [25–27]. Applied to clinicians’ thiazide prescribing behaviour, the TPB proposes that the most proximal antecedents of whether a clinician will perform a behaviour (in this case, prescribing thiazides) are their intention to perform the behaviour (whether they want to prescribe thiazides) and their perceived behavioural control (PBC) over the behaviour (whether they believe that they can prescribe thiazides). Their intention is in turn determined by three underlying cognitive constructs: their attitude (i.e., are they in favour or against prescribing thiazides), their subjective norm (their views of whether others think they should prescribe thiazides) and their PBC (see Fig. 1). A systematic review showed that studies using the TPB explained 59 % of the variance in intention and 35 % of the variance in health professional behaviour [27]. Behavioural theory has also been used to evaluate the process of trials of implementation interventions [19, 20, 28, 29] suggesting that behavioural theory may contribute to building a cumulative understanding of why implementation interventions are successful or not.

PEMs are a mode of delivering information. An implicit model might suggest a direct relationship between knowledge and behaviour change; however, such a model is not consistent with evidence [30]. The relationship between providing information and achieving behaviour change is more complex. When information is evidence-based and presented by a credible and influential source, it may be persuasive and could plausibly improve clinicians’ motivation to prescribe by altering their beliefs about consequences (e.g. attitude) and highlighting the social influences (e.g. subjective norm) of prescribing [23]. The TPB captures the motivational constructs that PEMs might plausibly influence [31]. Given the TPB’s evidenced predictive validity [27], it was arguably an appropriate model for evaluating the process of behaviour change in the pragmatic intervention tested in the OPEM trial.

That said, the TPB is not without its limitations and critics, and there is debate in some literatures about whether to retire the theory altogether [32, 33]. Furthermore, the TPB is not the only behavioural theory that could be selected to explain the process of behaviour change in implementation interventions [34]. Nevertheless, given the plausibility that PEMs may change behaviour through a motivational process, the TPB provides a replicable mediation model and measurement methods for understanding how behaviour change might operate through a motivational process in health professionals. Furthermore, using the TPB as a basis for process evaluation using an experimental design provides an opportunity to test the tenets of the theory itself, further contributing to the evidence to inform the utility of the theory moving forward. And irrespective of the theory itself, the TPB provides a potentially useful methodological exemplar for hypothesising and investigating mechanisms of action alongside trials of implementation interventions. We hypothesised that the OPEM intervention would be most likely to operate by changing physicians’ intention to prescribe thiazides to elderly patients due to improved attitude or subjective norm with little or no change in perceived behavioural control [16].

## Methods

### Summary of the main OPEM trial and OPEM TRY-ME sub-trial intervention

The detailed methods of the OPEM trial, the OPEM TRY-ME sub-trial and this theory-based process evaluation are available in the published protocols [16, 31, 35]. In summary, the OPEM trial was replicated three times for three different areas of quality improvement, and we conducted independent process evaluations of two of these replications of the OPEM trial. Results of the other replicate process evaluation are reported elsewhere [36]. The current paper describes the results of the OPEM trial testing PEMs aimed at increasing physicians’ prescription of thiazide diuretics for managing uncomplicated hypertension in patients ≥65 years old in Ontario, Canada.

Family physicians (FPs) in the OPEM trial were randomised to receive PEMs of differing levels of detail along with *informed*, an evidence-based newsletter mailed quarterly to approximately 15,000 Ontario FPs since 1994.

The OPEM trial team developed two forms of the PEMs, and a team of health psychologists independently developed a third version of the PEMs (the latter featuring in the OPEM TRY-ME sub-trial). The OPEM trial team developed long educational messages produced as a two-page insert into *informed* (indistinguishable from the rest of the periodical in size, style and editing) including background and evidence-based guideline and references. The OPEM trial team also developed short directive statements produced on a postcard-sized card stapled to the outside of *informed* as ‘outserts’, which were composed of brief information about the benefits of thiazides compared to other antihypertensive drugs. The trial team developed these outserts pragmatically (atheoretically*)* with a focus on clear communication of an actionable message, rather than a specific theory. In addition to these two PEMs, an independent team of health psychologists developed a third form of PEM: TPB-based outserts designed to address intention, attitude and subjective norm [31]. The ‘TPB outserts’ were similar in format (length, size, design) to the atheoretical outserts.

Using a 2 × 3 factorial design, FPs were randomised to receive either an *atheoretical outsert only*, a *TPB outsert only*, a *long insert only*, a *long insert and atheoretical outsert*, a *long insert and TPB outsert* only or a copy of the *informed* newsletter with no message (control condition).

The main OPEM trial results are reported elsewhere [11]. The current paper reports results of the process evaluation for the 2 × 3 factorial test of the effects of long inserts, short atheoretical outserts and short TPB outserts on changes in the TPB constructs (attitude, subjective norm and intention) that we hypothesised would explain change exerted by the intervention.

### Theory-based process evaluation study participants and sample size

Sampling and postal survey delivery proceeded as described in the protocol [16]. The OPEM trial team provided a sampling frame based on a random subset of physicians in the trial, excluding those sampled for the first process evaluation. We required 252 participants (63 per group) to have 80 % power of detecting an effect size of 0.5 standard deviations using a significance level of 5 %. We assumed a 50 % response rate for each survey (pre- and post-intervention), so our initial sample size was 1512 participants (252 per group).

### Questionnaire development

We developed questionnaires in accordance with standard methods [37] and the protocol [16]. We included a contextualising patient scenario, which preceded the TPB items and described a representative set of clinical and contextual features in which the targeted prescribing behaviour would typically take place, describing a patient with recurrent elevated blood pressure readings and unsuccessful attempts at controlling blood pressure with lifestyle modifications (see Additional file 1 for the scenario). The questionnaire included 18 items directly measuring TPB constructs scored on a seven-point Likert scale (see Additional file 1). The items were presented in random order except for attitude items, which were ordered consecutively to distinguish this construct’s response format (varying bipolar anchors) from the other TPB construct items. Each item was constructed using the Target, Action, Context and Time (TACT) principle [38] to specify the clinical behaviour under investigation. The Target was ‘this woman’ (referring to a description provided in the contextualising scenario); ‘prescribing thiazide diuretics’ was the Action; the Context was the 4th visit since her last annual physical (described in the scenario); and Time was (implicitly) during the consultation described in the scenario.

We supplemented TPB measures with context-specific items and self-reported past behaviour. A final open-ended question asked physicians ‘in your experience, what are the reasons (medical and non-medical) for which elderly patients may not be prescribed thiazide diuretics as a first-line treatment for their hypertension?’

### Data collection procedure

At baseline, we mailed the questionnaire using Dillman’s tailored design method for postal questionnaires [39]. We restricted the questionnaire to two pages in length and provided CDN$20 to every physician who returned a completed questionnaire. We sent pre-intervention questionnaires 8 weeks prior to disseminating the PEMs and post-intervention questionnaires to baseline respondents 6 months after dissemination of the PEMs.

TPB items were reverse-scored (where necessary) such that high scores represented agreement (or positive attitudes) and low scores, disagreement (or negative attitudes). We used the item mean scores within each theory-based construct (i.e. intention, attitude, subjective norm, and perceived behavioural control) to create a composite score for each construct. Three independent research assistants verified the accuracy of the data entry.

### Analyses

We assessed internal consistency (Cronbach’s alpha) on TPB constructs before creating mean scores for each construct. We used analyses of covariance to test the hypotheses and used a *p* value of 0.01 to adjust for multiple testing. We used a non-parametric analysis (Kruskal-Wallis test) to see whether results were robust against violations of assumptions. Low levels of missing data (<7 %) on TPB variables suggested that listwise deletion to handle missing data would be appropriate. We tested the appropriateness of the missing data strategy by re-running the analyses using two modified versions of the data: one which replaced all missing TPB values with data most supportive of our hypotheses and another replacing with data most opposed to our hypotheses.

We extracted demographic data from a physician database for a random sample of 20 % of respondents and non-respondents to the pre-intervention questionnaire to test for non-response bias. We used ANOVAs to assess the impact of attrition by comparing pre-intervention TPB of completers of pre- and post-intervention questionnaires to those who only completed pre-intervention surveys.

We conducted regression analyses with control group data to test the predictive utility and predictive pathways in the model. We regressed intention on attitude, subjective norm and perceived control (PBC) at baseline and regressed Time 2 self-reported behaviour on baseline intention and PBC.

Two researchers (JP, NR) coded all comments from the open-ended question (about perceived barriers that may prevent an elderly patient from being prescribed thiazides) such that two people coded each comment. Coding was compared and differences resolved through discussion, and then codes were grouped into themes reflecting the perceived source of each barrier.

## Results

### Response rate and non-response analysis

Six hundred and thirty-two (of 1512; 41.8 %) physicians returned the pre-intervention questionnaire, 468 (74.1 % of baseline, 31.0 % of total invited) of which also returned the post-intervention questionnaire (Table 1). Responder analysis showed that respondents were more likely to be affiliated with a university (9.2 vs. 2.1 %, *p* < 0.01) and more likely to be members of the College of Family Physicians of Canada (CFPC) (43.7 vs. 30.8 %, *p* < 0.05) than non-respondents (see Table 2). No differences were observed on baseline TPB constructs between those completing both time-points compared to those only completing baseline. Eligibility for inclusion in all subsequent analyses was defined as all respondents completing both pre- and post-intervention surveys with no missing data on any TPB variables (*N* = 426).

**Table 1:** Participant flow by group

	Groups					
	Insert only	Atheoretical outsert	TPB outsert	Insert and atheoretical outsert	Insert and TPB outsert	Control
Allocated and invited	252	252	252	252	252	252
Baseline return	96	104	98	110	106	118
Follow-up return	65	76	76	86	76	89
Excluded listwise (missing data)	9	4	6	6	8	9
Included in analysis	56	72	70	80	68	80

**Table 2:** Demographics comparison of baseline respondents and non-respondents

Demographic factor		Respondents	Non-respondents	Population^a^	Test results^b^
Graduating year	Mean	1978	1977	1981	*t*(263) = 0.329, *p* = 0.742
Sex	Male	77.3 %	78.8 %	63.0 %	*χ* ^2^(1, *N* = 265) = 0.081, *p* = 0.776
Urban/rural	Urban	89.1 %	91.1 %	91.9 %	*χ* ^2^(1, *N* = 265) = 0.302, *p* = 0.582
University affiliation	Yes	9.2 %	2.1 %	9.3 %	*χ* ^2^(1, *N* = 265) = 6.771, *p* = 0.009
CFPC member	Yes	43.7 %	30.8 %	46.7 %	*χ* ^2^(1, *N* = 265) = 4.684, *p* = 0.030

20 % random sample. *n* = 119 for respondents and *n* = 146 for non-respondents (four (respondents) and six (non-respondents) participants could not be found using MD Select and are thus not included in the analysis)

^a^Population based upon all physicians in Ontario specialising in either family medicine or physician/general practice (*N* = 10,429)

^b^Test results compare respondents to non-respondents

### Internal consistency

The Cronbach’s alpha for intention was 0.96 and 0.95 for pre- and post-intervention questionnaires (respectively), for subjective norm *α* = 0.90 and for attitude *α* = 0.86 for both pre- and post-intervention, and for PBC, α = 0.84 (pre-intervention) and 0.82 (post-intervention).

### Main effects of PEMs on intention, attitude, subjective norm and PBC

Physicians reported strong baseline intention to prescribe thiazides (overall mean = 5.93 out of 7; SD = 1.44; see Table 3 for details for each group). Controlling for pre-intervention intention, we did not observe a main effect for long insert, pragmatic outsert or TPB outsert PEMs on post-intervention intention to prescribe thiazides (primary outcome; see Table 4). On pre-intervention measures, physicians reported positive attitude (overall mean = 5.80, SD = 1.09), strong agreement with stated subjective norm (overall mean = 5.83, SD = 1.06) and strong agreement with statements of perceived behavioural control (overall mean = 6.20, SD = 1.01); see Table 2 for between group details. No significant main effects were observed for insert, pragmatic outsert or TPB outsert PEM groups on any of the three theory-based predictors of behavioural intention (secondary outcomes; see Table 5). The Kruskal-Wallis test also reflected these findings, as did analyses testing the appropriateness of listwise deletion as a missing data strategy. These null findings indicated that further mediation analyses as originally described in the protocol would be inappropriate as the first criterion for mediation was not met given that the intervention did not alter the theory-based mediators.

**Table 3:** Descriptive statistics for theory of planned behaviour constructs by group, before and after the OPEM trial

			Intention		Attitude		Subjective norm		Perceived behavioural control	
			Mean (sd)		Mean (sd)		Mean (sd)		Mean (sd)	
Groups		*N*	Baseline	Follow-up	Baseline	Follow-up	Baseline	Follow-up	Baseline	Follow-up
Insert	Atheoretical outsert	80	6.05 (1.38)	6.12 (1.19)	5.79 (0.98)	5.89 (0.96)	5.82 (1.11)	5.96 (0.93)	6.27 (0.91)	6.38 (0.83)
	TPB outsert	68	5.86 (1.62)	5.78 (1.57)	5.75 (1.16)	5.65 (1.19)	5.93 (1.02)	6.02 (0.97)	6.33 (0.93)	6.32 (0.99)
	No outsert	56	6.07 (1.29)	5.86 (1.46)	5.84 (1.11)	5.90 (1.12)	5.90 (1.00)	5.70 (1.12)	6.26 (0.90)	6.14 (1.14)
No insert	Atheoretical outsert	72	5.82 (1.51)	5.71 (1.53)	5.73 (1.15)	5.66 (1.12)	5.68 (1.02)	5.62 (1.15)	6.05 (1.09)	5.98 (1.09)
	TPB outsert	70	5.86 (1.43)	5.76 (1.58)	5.76 (1.18)	5.80 (1.14)	5.87 (1.10)	5.69 (1.17)	6.18 (1.07)	6.19 (1.08)
	No outsert	80	5.92 (1.44)	5.84 (1.59)	5.90 (0.99)	5.76 (1.17)	5.79 (1.08)	5.70 (1.18)	6.13 (1.13)	6.16 (1.03)

*TPB* theory of planned behaviour

**Table 4:** Results of analysis of covariance for primary outcome of change in intention (*N* = 426)

Effects	*F*	*p*	*B*	SE	95 % CI	
					Lower	Upper
Covariate						
Baseline Intention	231.37	<0.01	0.61	0.04	0.53	0.69
Main effects						
Insert PEM	0.46	0.50	0.08	0.12	−0.15	0.31
Outsert PEM (atheoretical)	0.42	0.52	0.09	0.14	−0.19	0.37
Outsert PEM (TPB)	0.01	0.95	−0.01	0.15	−0.30	0.28

*PEM* printed educational materials, *TPB* theory of planned behaviour

**Table 5:** Results of analysis of covariance for secondary outcomes (change in attitude, subjective norm, and perceived behavioural control) (*N* = 426)

TPB construct	Effects	*F*	*p*	B	SE	95 % CI	
						Lower	Upper
Attitude	Covariate						
	Baseline attitude	184.72	<0.01	0.61	0.04	0.53	0.69
	Main effects						
	Insert PEM	0.82	0.37	0.08	0.09	−0.09	0.25
	Outsert PEM (athoretical)	0.06	0.82	0.03	0.11	−0.19	0.24
	Outsert PEM (TPB)	0.03	0.86	−0.02	0.11	−0.23	0.20
Subjective norm	Covariate						
	Baseline subjective norm	80.10	<0.01	0.57	0.04	0.49	0.65
	Main effects						
	Insert PEM	3.69	0.06	0.17	0.09	−0.004	0.34
	Outsert PEM (athoretical)	1.45	0.23	0.13	0.11	−0.08	0.34
	Outsert PEM (TPB)	0.86	0.35	0.10	0.11	−0.11	0.32
Perceived behavioural control (PBC)	Covariate						
	Baseline PBC	127.08	<0.01	0.49	0.04	0.40	0.57
	Main effects						
	Insert PEM	1.20	0.27	0.10	0.09	−0.08	0.27
	Outsert PEM (athoretical)	0.11	0.74	0.04	0.11	−0.17	0.25
	Outsert PEM (TPB)	0.37	0.55	0.07	0.11	−0.15	0.28

### Testing the predictive efficacy of the TPB within the control group

In the control group,^1^ baseline attitude (*r* = 0.72, *p* < 0.01), subjective norm (*r* = 0.79, *p* < 0.01), and perceived behavioural control (*r* = 0.81, *p* < 0.01) were all strongly correlated with baseline intention. Baseline intention (*r* = 0.66, *p* < 0.01) and perceived behavioural control (*r* = 0.55, *p* < 0.01) both strongly correlated with follow-up self-reported behaviour. Residuals were plotted and were sufficiently normally distributed to proceed with interpretation. Regression of baseline intention scores on baseline TPB predictors of intention showed that attitude (*β* = 0.28, *p* < 0.01, *B* = 0.41, 95 % CI_*B*_ 0.19 to 0.64), subjective norm (*β* = 0.28, *p* < 0.05, *B* = 0.38, 95 % CI_*B*_ 0.09 to 0.68) and perceived behavioural control scores (*β* = 0.40, *p* < 0.01, *B* = 0.53 95 % CI_*B*_ 0.25 to 0.81) all significantly contributed to explaining 73.9 % of the variance (*R* ^2^_adj_) in baseline intention. Baseline intention (*β* = 0.63, *p* < 0.01, *B* = 1.27, 95 % CI_*B*_ 0.67 to 1.86) but not PBC (*β* = 0.04, *p* = 0.78, *B* = 0.11, 95 % CI_*B*_ = −0.68 to 0.90) predicted Time 2 self-reported behaviour, accounting for 42.6 % of its variance (*R* ^2^_adj_).

### Self-reported past behaviour

Physicians at baseline self-reported prescribing thiazides to a mean of 6.63 (*SD* = 2.71) of their 10 most recently seen elderly patients newly diagnosed with uncomplicated hypertension, suggesting potential scope for improvement and for greater consistency between physicians.

### Content analysis of perceived barriers to prescribing thiazides to elderly patients

Most physicians (95 % at baseline and 91 % at follow-up) provided at least one reason describing why elderly patients may not be prescribed thiazides as a first-line treatment for hypertension. We coded physicians’ responses and organised codes into themes representing the source of perceived barriers. ‘Patient clinical characteristics’ (‘Co-morbid conditions where other antihypertensives are better’, ‘Allergy to sulfonamides or thiazides’) were mentioned by most respondents to this question (75.7 % baseline, 73.4 % follow-up), followed by ‘patient comfort and side effects’ (‘Electrolyte disturbances; weak + dizzy + confusion’, ‘Increased urinary frequency as side effect’), mentioned by 62 % of physicians at baseline and 59.9 % at follow-up. 11.8 % of physicians at baseline and 10.6 % at follow-up described their ‘beliefs as not in favour of thiazides’ (“ACE Inhibitors offer benefits above and beyond BP control and are often a better 1st line choice”). A smaller percentage of physicians described other barriers, including ‘patient preference/adherence’ (‘Patient preference’), ‘system-related’ (‘Perception that diuretics are old and outdated drugs’, ‘Pharmaceutical pressure for more expensive drugs’), physician ‘beliefs in favour of thiazides’ (‘For this woman (Mrs. Kelly), I wouldn’t have many reasons not to prescribe a thiazide diuretic’) and ‘other’ (i.e. unique comments not fitting into other codes).

## Discussion

This study tested whether an information-provision intervention designed to promote guideline recommended prescribing of thiazides to elderly patients with uncomplicated hypertension operated by modifying family physicians’ attitudes, norms, control, and motivation to prescribe. The main OPEM trial itself did not observe any changes in thiazide prescription [11], and the intervention did not change theory-based determinants of thiazide prescription in this process evaluation. Nevertheless, the theoretical basis of this process evaluation provides a viable foundation for interpreting the trial’s null findings, which may inform and help to optimise future interventions. This process evaluation contributes to and builds on the growing literature on theory-based process evaluation [19, 20, 28, 29] by developing methods, employing them alongside the OPEM trial, and hypothesising a priori the process through which trial effects would operate.

At baseline, the sample of physicians reported strong intention (they wanted to prescribe thiazides), positive attitude (they believed prescribing thiazides is a good idea), high subjective norm (people important to them thought they should prescribe thiazides), and strong PBC (they believed that they could prescribe thiazides) over prescribing thiazides. Medium/large changes in intention are often reflected in small/medium changes in behaviour [40]; given how high the reported intention was in the process evaluation in all trial arms, we would expect that a ceiling effect precludes even a medium effect in the main trial. The theoretical basis suggests that PEMs were ineffective because they aimed to educate and persuade physicians about performing a behaviour that they already strongly intended to do.

In open-ended questions, family physicians highlighted patient-related clinical complications as reasons why elderly patients *may* not be prescribed thiazides, reflecting beliefs about negative consequences associated with prescribing to patients with complications or side effects. However, attitude towards prescribing was positive, suggesting these negative beliefs did not represent overall attitude towards prescribing thiazides and therefore may not be influencing motivation and behaviour. Such findings nevertheless suggest that future intervention to improve thiazide prescribing could involve patients’ views and behaviour as well.

Despite strong intention to prescribe, self-reported (6.63 of last 10 patients) and observed (28 % in main trial data) [11] thiazide prescription rates at baseline and follow-up did not reflect guideline recommendations. While prescription to every elderly patient with newly diagnosed uncomplicated hypertension may not always be appropriate and self-reported rates are likely an over-estimation of actual prescribing rates of thiazides, there nevertheless remains considerable room for improving thiazide prescription. This also suggests that while intention played a necessary role in prescribing thiazides, additional factors may have moderated the translation of these strong intentions into higher prescription rates. The TPB focuses largely on the predictors of intention (‘pre-intentional’ factors) but does not include any post-intentional factors to explain how intention is translated into action. This may have implications for interventions that only include techniques designed to increase motivation: had the OPEM interventions increased physicians’ intention to prescribe thiazides, post-intentional barriers may still have limited actual behaviour change had it been observed.

This process evaluation provides an inherent test of the TPB in two ways: predictive utility and explanation of behaviour change. We showed that the TPB’s predictive efficacy was consistent with systematic review evidence [27]. However, we could not assess whether the TPB explains behaviour change, as there was none to explain. Support for the TPB would require both a change in behaviour and a change in one or more TPB constructs, while evidence against the TPB would require a change in behaviour without any associated change in TPB constructs [16]; neither were possible here. Nevertheless, whether the TPB sufficiently captures all possible routes to behaviour change that a PEM may provide is debatable. There have been increasing calls from the literature to consider theories of behaviour that move beyond the TPB towards focusing on (in addition to attitudes, subjective norm, PBC and intention) post-intentional factors, habit, and automaticity [41] and the role of team and organisational factors [42, 43].

### Lessons learned and recommendations for conducting theory-based process evaluations

This process evaluation may help to advance methods of conducting theory-based process evaluations alongside RCTs of implementation interventions to help to understand the mechanism of effect (or lack thereof). We offer the following recommendations based on our experience. Theory-based process evaluations conducted alongside randomised controlled trials should in as far as is possible:

- *Reflect the trial’s design, including comparison and control group(s)*: For the OPEM process evaluation, this involved surveying a random sample of physicians from each of the main trial’s arms to meet the sample size requirements.
- *Collect data from trial participants pre- and post-intervention to control for baseline differences*: We surveyed physicians at two time points, before and 6 months after the intervention, in all trial arms. We showed this to be feasible, and the pre-test data demonstrated that there was little room for improvement in process measures from the start, which would not have been possible using a post-test only design.
- *Use previously tested theoretical models to provide consistency with the literature, generalizability, and foster a cumulative knowledge base*: A key purpose of a scientific theory is to summarise existing knowledge. By selecting an established theory with constructs that could plausibly explain the mechanism of action of an education-focused intervention, the findings can be compared against the existing literature, thereby facilitating future evidence syntheses and informing future PEM-based interventions.
- *Hypothesise the mediating mechanism a priori and conduct a mediation analysis if effects detected on primary and/or secondary trial outcomes*: We demonstrated how drawing upon a theory of behaviour provides the basis for hypothesising how the intervention’s mechanism of action might operate through this model. The challenge, particularly for interventions developed without a theory, remains in selecting and operationalizing a plausible theory for explaining the intervention effect. Whether the most plausible theory was selected in the present study is not clear given the lack of change in either process or outcome measures. Nevertheless, selecting a theoretical model that proposes a mediation pathway provides a basis for testing its mechanisms of action when possible [19].
- *Conduct formative theory-based investigations of determinants of the targeted behaviour prior to the trial*: The present study was conducted opportunistically alongside a pragmatic trial, and the findings helped to clarify why the intervention as specified was not effective in increasing thiazide prescribing. Ideally, the content of the PEMs might have been informed by formative research to investigate potential barriers and enablers [44]. While such approaches were not commonplace at the time the trial was conducted, future PEM-based interventions would benefit from assessing and addressing (in as much as is possible within the PEM-based method of delivery) the factors that may prevent change. Such formative investigations can also inform theory selection and early phase questionnaire research to ensure that there is ‘room for improvement’ in the anticipated mechanism of change prior to the trial and its process evaluation.
- *Assess the fidelity with which the intervention has been received, read, and responded to by the target participant*: In the present study, we could not assess the extent to which recipients read the PEMs, which could be an effect modifier. Building in fidelity assessment alongside mechanistic process evaluation is advised when possible.

This process evaluation provides a theoretical basis upon which future implementation interventions targeting increased thiazide prescription could draw instead of ‘going back to the drawing board’. In well-informed and motivated health professionals, if PEMs only deliver content targeting motivation, this may not be sufficiently potent. Instead, methods of delivering techniques that address post-intentional barriers to prescribing thiazides may be preferred. In such instances, theories of behaviour change such as the health action process approach [45] and dual process models [41, 46] that include and go beyond motivation would be more informative than motivational models such as the TPB.

A strength of this study is its use of a well-tested theory of behaviour operationalized according to best recommended practice to investigate the underlying mechanisms of an implementation intervention. By matching the 2 × 3 factorial design of the OPEM trial and assessing TPB cognitions before and after the intervention was delivered, we quantified existing levels of attitude, subjective norm, perceived behavioural control, and intention, providing an explanation for the lack of change in behaviour observed in the OPEM trial. While we showed strong internal consistency on all measures, some social desirability bias may have led to over reporting. However, this seems unlikely given the relatively low self-reported behaviour (mean six of last ten patients) and confidential nature of the data collection. Future research would benefit from assessing social desirability bias in questionnaires to health professionals. This study is also limited by an inability to link theory-based constructs to objective measures of behaviour from the main trial. This process evaluation is also limited by an observed response bias towards physicians who are part of a university and part of the College of Family Physicians of Canada, who may have had greater access and exposure to evidence prior to the OPEM trial and may have had more positive attitude and intention relative to the full trial sample. Our response rate was a further limitation, despite our evidence-based efforts to maximise recruitment to this study. However, the response rate was similar to other theory-based studies with health professionals and underscores the recognised challenge of recruitment and retention of health professionals in such studies. Our qualitative analyses helped to supplement our quantitative findings with additional contextual insight. Future theory-based process evaluations could be well served to also assess such contextual factors quantitatively to investigate whether they may operate as moderators of the intervention alongside the mediated mechanisms of change.

## Conclusions

By conducting a pre-post theory-based process evaluation matched to the factorial design of the main trial, this study advances the methodology of conducting process evaluations alongside randomised trials of implementation interventions and demonstrates the potential utility of drawing upon theory for interpreting the results of pragmatic trials. In this case, pre-existing strong intention, subjective norm, and positive attitude provide a theory-based explanation of why dissemination of printed educational materials may not result in a change in physicians’ prescribing behaviour. The use of printed educational materials for increasing prescription rates may therefore be ineffective when physicians’ pre-existing motivation to prescribe is strong. Future efforts at increasing prescription rates should consider targeting post-intentional factors.

## Additional file

Additional file 1: Electronic version of questionnaire. (PDF 89 kb)

## Footnotes

1
*n* = 76 analysed, four participants excluded due to missing data on follow-up self-reported behaviour
