# Supplementary material for: A theory-based process evaluation alongside a randomised controlled trial of printed educational messages to increase primary care physicians’ prescription of thiazide diuretics for hypertension [ISRCTN72772651]
Source: Implement Sci. 2016 Sep 13;11:121. doi: 10.1186/s13012-016-0485-4 (PMC5020459; doi:10.1186/s13012-016-0485-4)
Supplement: Additional file 1: — Electronic version of questionnaire. (PDF 89 kb) [file 13012_2016_485_MOESM1_ESM.pdf]

Please note: *If you do not wish to complete the questionnaire please send a blank one back to us so that we do not continue to send you reminder notices.*

Scenario: One of your patients, Mrs. Muriel Kelly, is visiting you today for the fourth time since her last annual physical which she had just over 6 months ago. Mrs. Kelly is 72 years old, happily married, with children who live out of town. She is retired and spends most of her time with volunteer work. She has no previous medical history and has not taken medication for any chronic disease in the past. She has had an annual flu shot every year since she turned 65. After having unsuccessfully attempted lifestyle changes during the previous 3 months, she is visiting you today to consider the initiation of an anti-hypertensive drug. Lab results are non contributory. She does not smoke. Her physical exam today is non contributory except for her blood pressure reading, which is 160/90 for the fourth time since her last annual physical. The attempt at lifestyle modification was ineffective in getting her blood pressure under control.

Please read each question carefully and answer it to the best of your ability. There are no correct or incorrect responses; we are merely interested in your point of view. The questionnaire may appear to be monotonous since several of the statements are worded in a repetitive manner. However, the scientific nature of the study requires this methodological approach. Your collaboration is vital. It is important to get <u>*your opinion*</u>.

I will prescribe thiazide diuretics to this woman as a first-line treatment of her hypertension.

Strongly agree 1 2 3 4 5 6 7 *Strongly disagree*

People who are important to me professionally think that I should prescribe thiazide diuretics to this woman as a first-line treatment of her hypertension.

Strongly agree 1 2 3 4 5 6 7 *Strongly disagree*

I am confident that I could prescribe thiazide diuretics to this woman as a first-line treatment of her hypertension.

Strongly agree 1 2 3 4 5 6 7 *Strongly disagree*

I have complete control over whether to prescribe thiazide diuretics to this woman as a first-line treatment of her hypertension.

Strongly agree 1 2 3 4 5 6 7 *Strongly disagree*

I think the Canadian Hypertension Education Program (CHEP) would approve of me prescribing thiazide diuretics to this woman as a first-line treatment of her hypertension.

Strongly agree 1 2 3 4 5 6 7 *Strongly disagree*

I think most general practitioners/family physicians would approve of me prescribing thiazide diuretics to this woman as a first-line treatment of her hypertension.

Strongly agree 1 2 3 4 5 6 7 *Strongly disagree*

I feel capable of prescribing thiazide diuretics to this woman as a first-line treatment of her hypertension.

Strongly agree 1 2 3 4 5 6 7 *Strongly disagree*

I plan to prescribe thiazide diuretics to this woman as a first-line treatment of her hypertension.

Strongly agree 1 2 3 4 5 6 7 *Strongly disagree*

I think the Heart and Stroke Foundation of Canada would approve of me prescribing thiazide diuretics to this woman as a first-line treatment of her hypertension.

Strongly agree 1 2 3 4 5 6 7 *Strongly disagree*

Code ___ ___ ___

There are factors outside of my control that would prevent me from prescribing thiazide diuretics to this woman as a first-line treatment of her hypertension.

Strongly agree	1	2	3	4	5	6	7	*Strongly disagree*

For me, prescribing thiazide diuretics to this woman as a first-line treatment of her hypertension would be:

Good practice	1	2	3	4	5	6	7	*Bad practice*
Helpful	1	2	3	4	5	6	7	*Unhelpful*
Necessary	1	2	3	4	5	6	7	*Unnecessary*
Satisfying	1	2	3	4	5	6	7	*Not satisfying*
Very easy	1	2	3	4	5	6	7	*Very difficult*

I think that other general practitioners/family physicians in Ontario would prescribe thiazide diuretics to this woman as a first-line treatment of her hypertension.

Strongly agree	1	2	3	4	5	6	7	*Strongly disagree*

I think the Ontario Medical Association (OMA) would approve of me prescribing thiazide diuretics to this woman as a first-line treatment of her hypertension.

Strongly agree	1	2	3	4	5	6	7	*Strongly disagree*

I intend to prescribe thiazide diuretics to this woman as a first-line treatment of her hypertension.

Strongly agree	1	2	3	4	5	6	7	*Strongly disagree*

In your view, what is the most effective first-line drug treatment for elderly patients presenting with uncomplicated hypertension, if lifestyle modification proves to be ineffective? (Check all that apply)

- ❑ ACE inhibitors
- ❑ Thiazide diuretics
- ❑ Beta blockers
- ❑ Angiotensin II receptor blockers (ARBs)
- ❑ Calcium channel blockers (CCBs)
- ❑ other ___________________

How important is the cost of antihypertensive drugs in your choice of first-line drug treatment for elderly patients presenting with uncomplicated hypertension?

Very important	1	2	3	4	5	6	7	*Not at all important*

What sources of information (or who) would you consult for clinical decision making around management of hypertension?

____

____

Thinking about your last 10 elderly patients newly diagnosed with uncomplicated hypertension, for how many of them did you prescribe thiazide diuretics as a first-line drug treatment? ____ of 10

In your experience, what are the reasons (medical **and** non-medical) for which elderly patients may **not** be prescribed thiazide diuretics as a first-line treatment for their hypertension?

____

____

____

____

____

____

Thank you for your time

Please fax back to **XXX XXX-XXXX** or mail using the stamped self-addressed envelope included in this package.
